# Treatment of malaria restricted to laboratory-confirmed cases: a prospective cohort study in Ugandan children

**DOI:** 10.1186/1475-2875-6-7

**Published:** 2007-01-21

**Authors:** Denise Njama-Meya, Tamara D Clark, Bridget Nzarubara, Sarah Staedke, Moses R Kamya, Grant Dorsey

**Affiliations:** 1 Makerere University Medical School, Kampala, Uganda; 2 Department of Medicine, University of California San Francisco, USA; 3 London School of Hygiene and Tropical Medicine, London, UK

## Abstract

**Background:**

Presumptive treatment of malaria in febrile children is widely advocated in Africa. This may occur in the absence of diagnostic testing or even when diagnostic testing is performed but fails to detect malaria parasites. Such over-treatment of malaria has been tolerated in the era of inexpensive and safe monotherapy. However, with the introduction of new artemisinin-based combination therapy (ACT), presumptive treatment becomes economically and clinically less acceptable.

**Methods:**

The risks and benefits of only treating children with microscopy confirmed malaria using a prospective cohort design were investigated. A representative sample of 601 children between one and 10 years of age were recruited from a census population in Kampala, Uganda and were followed for all of their health care needs in a study clinic. Standard microscopy was performed each time a child presented with a new episode of fever and antimalarial therapy given only if the blood smear was positive.

**Results:**

Of 5,895 visits for new medical problems 40% were for febrile illnesses. Of the 2,359 episodes of new febrile illnesses, blood smears were initially reported as negative in 1,608 (68%) and no antimalarial therapy was given. Six of these initially negative smears were reported to be positive following quality control reading of all blood smears: four of these patients were subsequently diagnosed with uncomplicated malaria and two cleared their parasites without antimalarial treatment. Of the 1,602 new febrile illnesses in which the final blood smear reading was classified as negative, only 13 episodes (0.8%) were diagnosed with malaria in the subsequent 7 days. All 13 of these episodes of malaria were uncomplicated and were successfully treated.

**Conclusion:**

In this urban setting, malaria was responsible for only 32% of febrile episodes. Withholding antimalarial therapy in febrile children with negative blood smears was safe and saved over 1,600 antimalarial treatments in 601 children over an 18-month period. In the era of expensive ACT, directing resources towards improving diagnostic and treatment practices may provide a cost-effective measure for promoting rational use of antimalarial therapy.

## Background

The practice of presumptive treatment of malaria has been widely advocated in sub-Saharan Africa as a means of increasing antimalarial coverage and reducing the risk of progression to severe disease and death. However, it is well known that such practice frequently results in the unnecessary use of antimalarials, especially in lower transmission settings and older patient populations [1,2]. Such over-treatment of malaria has been tolerated in the era of inexpensive and safe antimalarial drugs such as chloroquine. However, due to the spread of drug resistance, many African countries are moving to artemisinin-based combination therapy (ACT) where presumptive treatment becomes economically and clinically less acceptable. In this new era of ACT, there has been an increased emphasis on antimalarial treatment based on laboratory confirmation [3].

Microscopy remains the gold standard for laboratory confirmation of malaria and its use could provide an efficient and cost effective means of significantly reducing the number of unnecessary antimalarial treatments [4]. However, there is evidence that the results of microscopy may have limited impact on antimalarial treatment practices even when the blood smear is negative [5,6]. Indeed, the most recent antimalarial treatment guidelines from Uganda recommend "any patient with fever or history of fever within the last 24 hours without evidence of other diseases should be treated for malaria even with a negative blood smear for malaria parasites" [7]. One potential factor contributing to the practice of prescribing antimalarial therapy in the setting of a negative blood smear is the belief that patients may still have malaria and lack of antimalarial treatment will lead to a poor outcome. In this study, we investigated the risks and benefits of giving antimalarial treatment only when confirmed by microscopy in a cohort of 601 Ugandan children aged 1–10 years living in a meso-endemic area followed prospectively for all of their health care needs over an 18 month period.

## Methods

### Description of study site and recruitment of study participants

This study was conducted in Kampala, the capital of Uganda, with an estimated population of 1.2 million. Malaria is meso-endemic in this area (parasite prevalence 17% among children) [8], occurring throughout the year with two peaks during the rainy seasons from March-May and September-November. Data from this study comes from a representative cohort of 601 children aged 1 to 10 years recruited as part of a primary longitudinal clinical trial of antimalarial therapy. Details of the screening and recruitment of this cohort have been published previously [9]. Briefly, a census was performed in a geographically defined urban slum adjacent to our study clinic. Children were recruited from this census population using probability sampling if they fulfilled all of the following eligibility criteria: 1) age 1 to 10 years, 2) agreement to come to the study clinic for any febrile episode or other illness, 3) agreement to remain in Kampala for the duration of the study, 4) agreement to avoid medications administered outside the study protocol, 5) weight ≥ 10 kg, 6) absence of severe malnutrition, 7) absence of known serious chronic diseases requiring frequent medical attention (e.g. AIDS, sickle cell disease, malignancy), 8) absence of life-threatening screening laboratory results, and 9) willingness of parents or guardians to provide informed consent. Enrollment took place between November 2004 and April 2005.

### Follow-up of study participants

Participants were followed up at a designated study clinic located in the outpatient department of Mulago Hospital, the primary referral hospital in Uganda. Parents/guardians were asked to bring their child to the study clinic for all of their medical care. The study clinic was open daily from 8:00 am to 5:00 pm and after-hours care was available at Mulago Hospital.

Children who presented to the study clinic with new medical problems underwent a standardized medical evaluation. Subjects who were febrile (tympanic temperature ≥ 38.0°C) or reported history of fever in the previous 24 hours had blood obtained by fingerprick for a thick blood smear. If the thick blood smear was positive, the patient was diagnosed with malaria regardless of the parasite density. Patients with uncomplicated malaria were given directly observed therapy with one of three combination antimalarial regimens under evaluation. Patients with severe malaria [10] or danger signs [11] were treated with quinine. Patients diagnosed with malaria and other concomitant febrile illnesses were treated for both. If the thick blood smear was negative the patient was not given antimalarial therapy and managed at the discretion of the study physicians. Standardized treatment algorithms were developed to help guide therapy for non-malarial illnesses. Medications with antimalarial activity were avoided for the treatment of non-malarial illnesses, including tetracyclines, antifolates, and macrolide antibiotics, when acceptable alternatives are available. Antihelminths and Vitamin A were routinely prescribed as per the Integrated Management of Childhood Illnesses (IMCI) guidelines. When necessary, participants were referred to the appropriate facility within the Mulago Hospital complex.

Study participants were encouraged to visit the Mulago Hospital pediatric emergency department when urgent care was needed outside of study clinic hours. Participants were instructed to inform emergency department personnel of their involvement in the study at the time of registration and to visit the study clinic on the following day. Identified Mulago Hospital personnel were educated about the study protocol and a nurse, employed by the project, oversaw adherence to the study protocol.

Children were evaluated for routine assessment if approximately 30 days elapsed without having a blood smear obtained. These routine assessments were made to ensure compliance with the study protocol and obtain blood smears for the evaluation of asymptomatic parasitaemia.

### Microscopy

Thick blood smears were stained with 2% Giemsa for 30 minutes. Parasite density was estimated by counting the number of asexual parasites per 200 white blood cells and calculating parasites per μL, assuming a white blood cell count of 8,000 cells per μL. A smear was judged to be negative if no parasites were seen after review of 100 high powered fields. The diagnosis and management of malaria was based on initial readings of blood smears. Final microscopy results were based on a rigorous quality control system which included a second microscopist re-reading all blood smears and any discrepancies between the first and second readings resolved by a third microscopist.

### Ethical approval and data management

Written informed consent was obtained from the parents/guardians of children for their participation in the study. Ethical approval was obtained from the Uganda National Council of Science and Technology, the Makerere University Research and Ethics Committee, and the University of California, San Francisco Institutional Review Board.

Data were double-entered in Access (Microsoft Corporation, Redmond, WA) and statistical analysis was performed using Stata version 8 (Stata, College Station, TX, USA). Categorical variables were compared using the chi-square test. A p-value of < 0.05 was considered statistically significant.

## Results

### Baseline characteristics of the study participants

A detailed description of the baseline characteristics of the 601 children enrolled in the cohort have been published [9]. Briefly, the mean age was 5.8 years, 37% were under the age of five, and 48% were female. Any bednet use was reported in 43% of the cohort, but only 6% reported using an insecticide-treated bednet (ITN). The prevalence of asymptomatic parasitaemia was 19% and 3% of children were found to have symptomatic malaria (fever and positive blood smear) on the day of enrollment.

### Follow-up of study participants

A total of 77 children were prematurely excluded from the study for the following reasons; movement out of Kampala for > 60 days (n = 49), inability to locate child for > 60 consecutive days (n = 12), withdrawal of consent (n = 11), and diagnosis of serious chronic disease requiring frequent medical care (n = 5). Follow-up for the remaining 524 patients continued until May-June 2006, at which time ITNs were distributed to the entire cohort. The cumulative follow-up period for the cohort was 740 person years, which constituted 93% of the potential follow-up time.

### Study clinic visits for new medical problems

**Figure 1:**
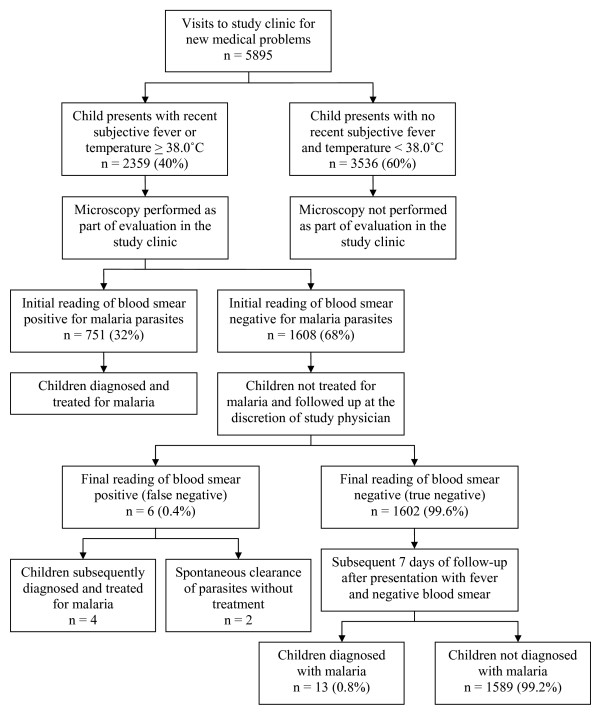
**Profile of visits made to the study clinic for new medical problems**. Initial reading of blood smear defined as the first reading done in the study clinic that was used for clinical decision making. The final reading of blood smear included reading by a second microscopist and reading by a third microscopist if there were discrepancies between the first and second readings.

A total of 5,895 visits were made to the study clinic for new medical problems. Among all visits for new medical problems, 2,359 (40%) were for febrile illnesses (tympanic temperature ≥ 38.0°C and/or subjective fever in the previous 24 hours) (Figure 1). A total of 59 children (10%) never experienced a febrile episode. In the remaining 542 participants, the median number of visits for new febrile illnesses was 4 (range 1–16).

Of the 2,359 episodes of new febrile illnesses, 751 (32%) were associated with an initial positive blood smear, and the participants were diagnosed and treated for malaria (Figure 1). None of the 751 initial positive blood smears were found to be negative after quality control reading (specificity of initial blood smear reading = 100%). A total of 270 children (45%) had no episodes of malaria during the study follow-up. In the remaining 331 participants, the median number of episodes of malaria was 2 (range 1–12).

### Outcomes of febrile illnesses with blood smears negative for malarial parasites

In a total of 1,608 episodes of febrile illnesses the initial blood smear was read as negative and treatment for malaria was not given. Six of these blood smears were subsequently determined to be positive for malarial parasites after quality control reading, giving a sensitivity of 99.6% for the initial blood smear reading (Figure 1). Two of the patients with false negative initial blood smears readings had very low parasite densities (16 asexual parasites/μL) and spontaneously cleared their parasites without antimalarial treatment. Two of the patients with false negative blood smears returned the following day with fever and parasitaemia and were treated successfully for uncomplicated malaria. In the remaining two patients with false negative blood smears, the fever resolved but parasitaemia persisted until the time they were diagnosed and successfully treated for uncomplicated malaria 51 and 61 days after the false negative blood smear reading, respectively.

To assess whether febrile illnesses were possibly due to sub-patent parasitaemia, the risk of being diagnosed with malaria was assessed over the subsequent seven days in the 1,602 febrile episodes with negative blood smears (Figure 1). A total of 13 episodes (0.8%) of new febrile illnesses with negative blood smears were diagnosed with malaria in the subsequent seven days. All 13 of these episodes of malaria were uncomplicated and successfully treated. For 10 of these 13 events, the initial illness reportedly resolved and a new illness had occurred at the time malaria was diagnosed three to six days later, suggesting malaria was not the cause of fever at the time the blood smear was initially read as negative. In the remaining three events, the initial febrile illness had persisted up until the time malaria was diagnosed one to five days later, suggesting malaria was the cause of the fever at the time the blood smear was initially read as negative. The incidence of malaria in the seven days following a negative smear in a febrile patient was significantly lower than the incidence of malaria over the entire course of the study (0.42 vs. 1.01 per person year, p = 0.0003).

### Diagnosis and treatment of non-malarial febrile illnesses

Data on physicians' diagnoses and treatments for all non-malarial febrile illnesses based on standardized guidelines developed locally were systematically collected. Diagnoses included a wide range of common childhood illnesses. The most common illnesses were upper respiratory tract infection, common cold, and non-specified fever (Table 1). Pneumonia, urinary tract infection, and otitis media occurred less frequently. Younger children (less than 5 years of age) were more likely to have pneumonia, diarrhea, or otitis media as a cause of their fever compared to those 5 years or older. In contrast, febrile illness were more likely to be non-specified (without localizing signs or symptoms and primary investigations yielded negative results) in older children. Most of the treatments prescribed for nonmalarial febrile illnesses were for the management of symptoms, including paracetamol, cough syrup, and vitamin C (Table 2). Less than half (45%) received an antibiotic, with amoxicillin being the most commonly prescribed. Young children were more likely to receive antibiotics compared to older children (56% vs. 44%, p < 0.0001). An antibiotic was prescribed in 96% of patients when the febrile illness was due to pneumonia, urinary tract infection, or otitis media. In contrast, only 45% of patients with febrile illnesses due to upper respiratory tract infections or common colds received antibiotics and only 28% of patients with non-specified febrile illnesses received antibiotics.

**Table 1:** The most common diagnoses of non malarial febrile illnesses

Diagnoses*	***Total (%) n = 1602***	***Age < 5 years (%) n = 742***	***Age ≥ 5 years (%) n = 860***	***RR^***† ***^(95%CI)***	***p-value***
Upper respiratory tract infection	754 (47%)	388 (52%)	366 (43%)	1.2 (1.1–1.4)	0.0001
Common cold	469 (29%)	247 (33%)	222 (26%)	1.3 (1.1–1.5)	0.001
Non-specified fever	233 (15%)	86 (12%)	147 (17%)	0.7 (0.5–0.9)	0.002
Pharyngitis/Tonsillitis	196 12%)	76 (10%)	120 (14%)	0.7 (0.6–1.0)	0.028
Diarrhea	157(10%)	95 (13%)	62 (7%)	1.8 (1.3–2.4)	0.0002
Skin infections/wounds	121 (8%)	55 (7%)	66 (7%)	1.0 (0.7–1.4)	0.843
Pneumonia	57(4%)	43 (6%)	14 (2%)	3.6 (2.0–6.5)	<0.001
Urinary tract infection	32 (2%)	15 (2%)	17 (0.8%)	1.0 (0.5–2.0)	0.949
Otitis media	22 (1%)	17 (2%)	5 (0.6%)	3.9 (1.5–10.6)	0.003

* Patients may have had more that one diagnosis

^† ^RR = Relative risk for age < 5 years versus age ≥ 5 years

**Table 2:** The most common treatments prescribed for non-malarial febrile illnesses

Treatments*	***Total (%) n = 1602***	***Age < 5 years (%) n = 742***	***Age ≥ 5 years (%) n = 860***	***RR^***† ***^(95%CI)***	***p-value***
Paracetamol	1448 (90%)	655 (88%)	793 (92%)	1.0 (1.0–1.2)	0.003
Cough syrups	767 (48%)	394 (53%)	373 (43%)	1.2 (1.1–1.4)	0.0001
Vitamin C	696 (43%)	363 (49%)	333 (39%)	1.3 (1.1–1.4)	<0.001
Amoxicillin	457 (29%)	233 (31%)	224 (26%)	1.2 (1.0–1.4))	0.018
Other antibiotics	332 (21%)	181 (24%)	151 (18%)	1.4 (1.1–1.7)	0.001
Oral rehydration salts	199 (12%)	111 (15%)	88 (10%)	1.5 (1.1–1.9)	0.004
Vitamin A	63 (4%)	35 (5%)	28 (3%)	1.5 (0.9–2.4)	0.134

* Patients may have been given more than one treatment

^† ^RR = Relative risk for age < 5 years versus age ≥ 5 years

### Risk of severe illnesses

The most important goal of malaria case management is to prevent the progression to severe disease and death. In this study we were able to estimate the risk of severe disease due to malaria and nonmalarial illnesses in a cohort of Ugandan children receiving free comprehensive care where malaria was only treated if confirmed by microscopy. There were no deaths during the study and no episodes of severe malaria based on standardized WHO criteria [10]. There were 16 episodes of malaria accompanied by danger signs [11] and 3 episodes with hyperparasitaemia (> 500,000 asexual parasites/μL) that were hospitalized and treated with quinine in accordance with our study protocol. Danger signs included single convulsions (n = 12), inability to sit up or stand (n = 2), persistent vomiting (n = 1) and lethargy (n = 1). An additional 22 hospitalizations occurred due to non-malarial illnesses that included the following; pneumonia (n = 6), fractures (n = 2), pyomyositis (n = 2), fever of unknown origin (n = 2), abscess drainage (n = 1), sinusitis (n = 1), febrile convulsions (n = 1), cellulitis (n = 1), severe weakness of unknown etiology (n = 1), asthma (n = 1), lead poisoning (n = 1), chickenpox (n = 1), diarrhea (n = 1), and tonsillitis (n = 1).

## Discussion

In this study, the risks and benefits of giving antimalarial treatment for febrile illnesses only when parasitaemia was confirmed by microscopy were assessed in a cohort of 601 children aged 1–10 years followed over an 18-month period. Minimal risks of withholding antimalarial treatment in over 1,600 episodes of febrile illnesses with negative blood smears were observed. In only four cases were initial blood smears falsely read as negative and children subsequently diagnosed with uncomplicated malaria. In another 13 cases, febrile illnesses were possibly due to sub-patent parasitaemia where children with truly negative blood smears were diagnosed with uncomplicated malaria over the subsequent seven days. Thus, only 1% of cases of febrile illnesses not treated for malaria would have likely benefited from antimalarial therapy and there was no evidence that a delay in therapy lead to any serious adverse outcomes. In contrast, the benefits of withholding antimalarial therapy in febrile children with negative blood smears were substantial. The numbers of antimalarial treatments were reduced from 2,359, assuming all febrile episodes would have been treated presumptively, to the 751 cases where malaria was confirmed by microscopy. Not only did this reduce the risk of adverse consequences associated with unnecessary antimalarial therapy but allowed clinicians to focus on the true causes of fever, which were generally mild and self-limiting.

The policy of presumptive treatment of malaria for all febrile illnesses has been widely advocated in sub-Saharan Africa, especially in young children. Efforts have been made to improve the rationale delivery of presumptive antimalarial therapy through the use of clinical algorithms with limited success [1,12,13]. Thus in the absence of laboratory confirmation, presumptive antimalarial therapy will always be a balance between the potential benefits of maximizing antimalarial coverage with the risks of providing unnecessary therapy. In the era of inexpensive and safe therapy with chloroquine, it was deemed economically and clinically acceptable to maximize coverage through the promotion of presumptive therapy. However, many African countries are currently in the process of changing to ACT as first-line treatment for malaria due to the spread of resistance to chloroquine and other inexpensive mono-therapies. ACT has been shown to be highly effective, but due to their increased cost and limited availability there has been an increased interest in restricting treatment of malaria to laboratory confirmed cases as a means of promoting rationale drug use [3].

Light microscopy offers an inexpensive and practical means of diagnosing malaria. In order to maximize its impact on antimalarial treatment practices, microscopy must be available and utilized appropriately, results must be accurate, and health care providers must use microscopy results to guide antimalarial treatment practices in a rationale way. In this study we did not address the issue of availability or utilization of microscopy as all febrile children were referred for microscopy. In this setting the diagnostic accuracy of microscopy was excellent, with a sensitivity of 99.6% and a specificity of 100%. This excellent microscopy was attributed to having access to good microscopes, quality staining material, and most importantly, highly experienced microscopists. Other studies from Africa in non-research settings have demonstrated that the diagnostic accuracy of microscopy may not reach the level obtained in our study, but can achieve acceptable results. A study done at six health centers in Zambia, reported fairly good results with an overall sensitivity of 88% and specificity of 91% [6]. In contrast, recent results from two government health facilities in Kenya reported an overall sensitivity of 69% and specificity of 62% when comparing initial microscopy readings to expert microscopy readings [14]. These findings underline the importance of the quality of routine microscopy. Results must have an acceptable level of accuracy in order for health care providers to have the confidence to use these results to make decisions about antimalarial therapy. A national laboratory survey in Ghana demonstrated that most laboratory staff lacked professional qualifications and there were marked regional differences in essential resources for malaria diagnosis [15]. Following a national laboratory training programme, researchers were able to demonstrate a modest improvement in the number of laboratories producing microscopy results of acceptable quality [15].

If a blood slide is requested it is logical to expect the results to guide treatment decisions. In this study all positive blood smears from febrile children were treated for malaria. There is little controversy regarding the practice of giving antimalarial therapy to symptomatic patients with positive blood smears. However, there are reports of up to 30% of patients with confirmed malaria not given antimalarial treatment in rural Tanzania [16]. Such findings raise the question of whether blood smear results were even being used to influence treatment practices. A more frequent problem is the practice of giving antimalarial therapy when a blood smear is reported as negative, especially in low transmission areas and in older patient populations. In a cross-sectional study from Kenya 79% of patients over the age of five years with a reported negative blood smear were given antimalarial therapy [14]. Similar results have been reported from a low transmission area of Tanzania where 48% of patients with negative blood smears were given antimalarial therapy [5] and in Zambia where 35% of smear negative patients were treated for malaria [6]. This practice is likely driven by the common belief that a patient may still have malaria even when the blood smear is negative, a position which has been promoted by national [7] and international guidelines [17]. In this study, febrile children with negative blood smears were followed prospectively and their outcomes clearly showed that there is almost no risk associated with not providing antimalarial therapy in our setting. The practice of not giving antimalarial therapy to patients with negative blood smears has the additional benefit of encouraging health care providers to diagnose the true cause of their illness. If the true cause of fever is not appropriately diagnosed, this may result in a delay in introducing appropriate treatment, especially antibiotics for bacterial infections, leading to an increased risk of severe complications and death [18]. The diagnosis of malaria is often convenient and provides a simple choice for therapy. Further research is needed in developing simple diagnostic and treatment algorithms that can be applied to patients with non-malaria fevers in resource limited settings of Africa.

## Conclusion

In the era of expensive artemisinin combination therapy, there is a great need to direct resources toward promoting the rationale delivery of antimalarial therapy by improving diagnostic and treatment practices in Africa. In this setting, where malaria transmission was relatively low, the practice of treating febrile children for malaria only when the diagnosis was confirmed by microscopy was a safe and highly effective means of reducing the number of antimalarial treatments given. The practice of providing antimalarial therapy when the blood smear is negative in this patient population is strongly discouraged. In addition, similar prospective studies to investigate whether such recommendations are appropriate for other epidemiological settings are encouraged.

## Authors' contributions

DNM, GD MK, SS, and TC conceived and designed the study. BN, DNM, and TC participated in data collection. DNM, TC, and GD participated in the data analysis. All authors participated in the writing of the manuscript, read and approved the final manuscript.

## Financial support

The study received financial support from the National Institutes of Allergy and Infectious Disease (AI052142).
